# Obesity Is a Risk Factor for Renal Dysfunction Following Persistent Proteinuria: An Observational Cohort Study

**DOI:** 10.31662/jmaj.2021-0223

**Published:** 2022-03-11

**Authors:** Kei Nagai, Masami Nakajima, Shinichi Yoshii

**Affiliations:** 1 Department of Nephrology, Hitachi General Hospital, Hitachi, Japan; 2 Department of Nephrology, Faculty of Medicine, University of Tsukuba, Tsukuba, Japan; 3 Hitachinaka General Hospital, Hitachinaka, Japan

**Keywords:** chronic kidney disease, obesity, persistent proteinuria

Obesity has been identified as a lifestyle-related disease, and its prevalence has seen a steady increase for the past years. Moreover, obesity has been associated with the risk for chronic kidney disease (CKD), which is defined as persistent proteinuria and/or renal dysfunction for more than 3 months ^(1)^. However, the clinical significance of obesity-related renal involvement needs a longer observation period compared to primary glomerular diseases, as pathological renal dysfunction is known to occur as an end-stage result of persistent glomerular overfiltration and proteinuria ^(2)^. In epidemiological studies, whereas most of the results showed that obesity is a risk factor both for proteinuria and for renal dysfunction, the causality between them in each individual case was disregarded. Thus, in this study, we aim to explore whether obesity may precede renal dysfunction as a result of proteinuria using the data from annual health checkups held in a Japanese facility with long-term follow-up from 1988 to 2021.

In total, 529,437 person-years (N = 54,883) of health checkup data were examined in this study. To accurately determine the incidence of proteinuria and the incidence of a reduced estimated glomerular filtration rate (eGFR), persons with negative results on the first and second examinations were included for analysis (Figure 1). Persistency of proteinuria and reduced eGFR was judged on consecutive positive results. Based on this algorithm, 313 patients with newly developed proteinuria and 3,676 with low eGFR less than 60 mL/min/1.73 m^2^ were identified. Renal dysfunction following persistent proteinuria was considered as the occurrence of reduced GFR after persistent proteinuria, which may be contrasted to incident persistent proteinuria as a result of renal dysfunction (Figure 1). Figure 2 shows the time course of 123 subjects with both persistent proteinuria and reduced eGFR. New onset of persistent proteinuria was observed to occur 5.8 ± 5.0 years after reduced eGFR in 43 subjects, while new onset of low eGFR occurred 5.8 ± 5.0 years after incident persistent proteinuria in 64 subjects (Figure 2). Furthermore, to characterize proteinuric renal dysfunction with any causality, subgroups were categorized as no renal event during follow-up, low eGFR alone, proteinuria alone, proteinuria following low eGFR, low eGFR following proteinuria, and simultaneous incidence of proteinuria and low eGFR (Table 1). Compared to subjects without any renal events, weight, fasting blood sugar level, systolic blood pressure, and diastolic blood pressure were noted to be globally higher in those with both proteinuria and low eGFR. It was also highlighted that the age of those with proteinuria following low eGFR and the body mass index (BMI) of those with low eGFR following proteinuria were significantly higher (Table 1). Aging has been identified as one of the definitive risk factors for renal dysfunction because the physiological changes between the fifth and sixth decades are known to affect glomerular and tubular function, systemic hemodynamics, and general homeostasis ^(3)^. As shown in Table 2, on multivariable adjusted logistic regression analyses, aging was found to be a risk factor for incident low eGFR alone (odds ratio [95% confidence interval], 1.019 [1.014-1.023] for 1 year) and proteinuria following low eGFR (1.047 [1.009-1.086]). On the other hand, age was not a risk factor for outcomes related to predominantly incident proteinuria, such as for proteinuria alone (1.000 [0.986-1.017]) and for low eGFR following proteinuria (1.000 [0.972-1.030]), suggesting that renal dysfunction as a result of persistent proteinuria occurred independent of age. Since the BMI and health outcomes generally have a U-shaped relationship ^(4)^, a dummy variable based on the obesity definition by the Japan Society for the Study of Obesity was set. Normal weight is defined as a BMI of 18.5 kg/m^2^ or more and less than 25 kg/m^2^; unhealthy “underweight” is defined as a BMI less than 18.5 kg/m^2^; and “obesity” is defined as a BMI of 25 kg/m^2^ or more ^(5)^. In predominant incident proteinuria, obesity is considered a significant risk factor for incident proteinuria alone (odds ratio [95% confidence interval], 1.418 [1.024-1.964]) and low eGFR following proteinuria (1.993 [1.172-3.388]), compared to the reference (normal weight: BMI 18.5-25.0 kg/m^2^). Collectively, these results show that persistent proteinuria and persistent renal dysfunction, representing CKD, can have different risk factors, with age for low eGFR and baseline obesity for proteinuria.

**Figure 1.**
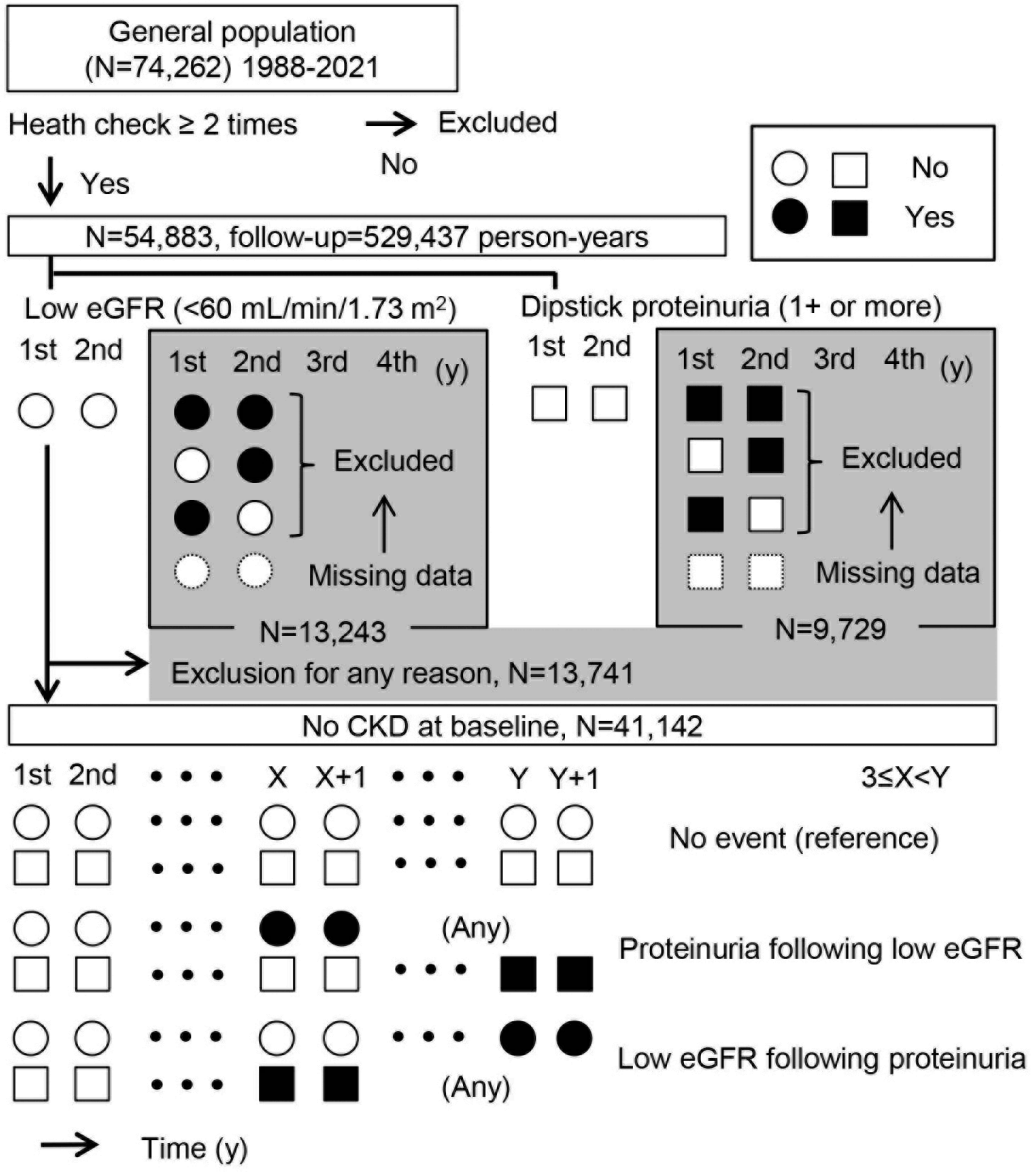
Design of the observational study over 30 years: 1988-2021. Participants in more than two annual health checkups were selected. Among them, subjects with any positive results in the first and second years were excluded. To determine the incidence of persistent proteinuria and a low estimated glomerular filtration rate (eGFR), persistency was judged based on consecutive positive results in the following years.

**Figure 2.**
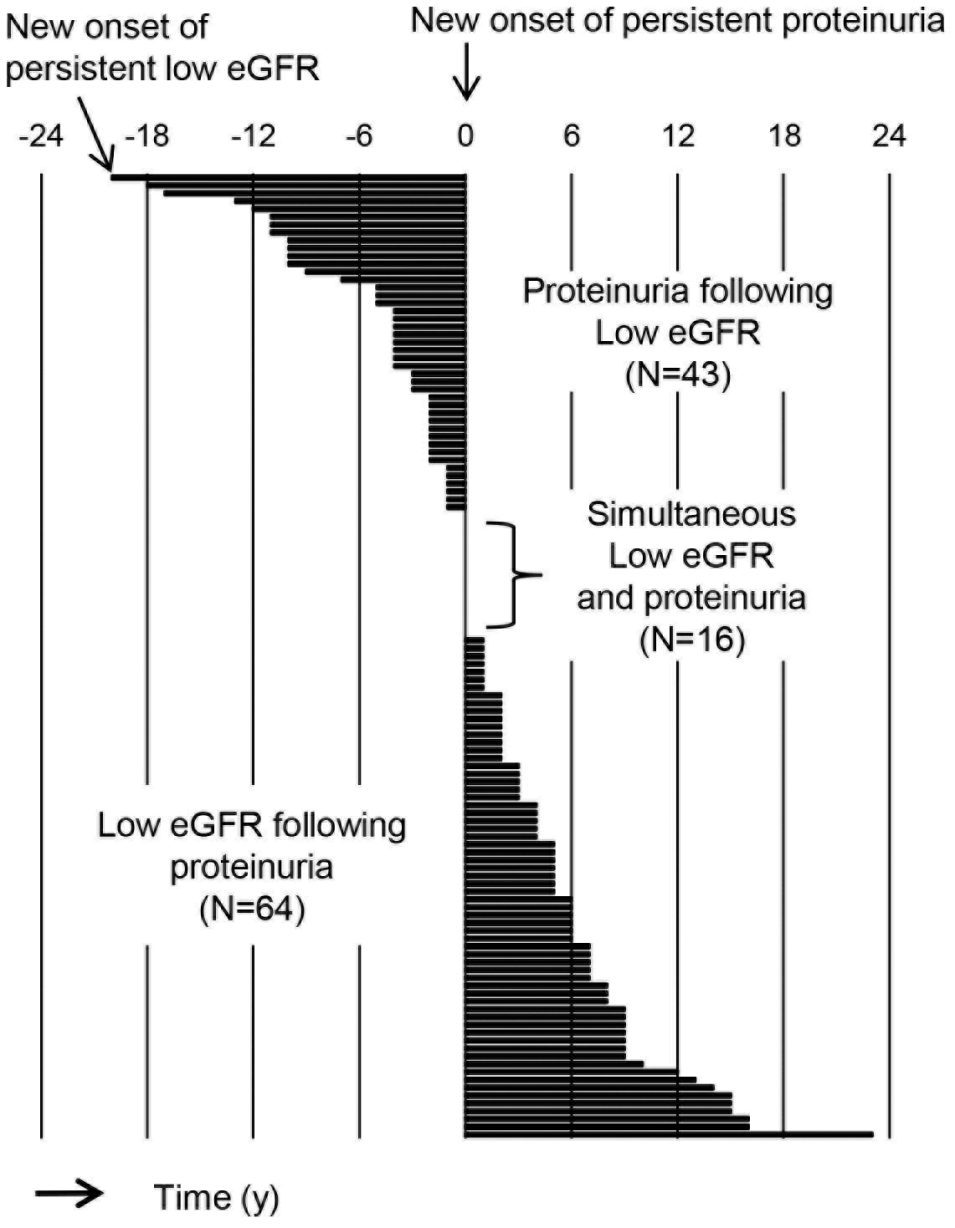
Time course of 123 subjects with both persistent proteinuria and persistent low eGFR. Proteinuria was defined as 1+ or more on dipstick urine test results, while low estimated glomerular filtration rate (eGFR) was defined as less than 60 mL/min/1.73 m^2^. A bar represents time (years) between new onset of persistent proteinuria and that of persistent low eGFR in each individual.

**Table 1.** Baseline Data of the Subjects with and without Renal Events.

	No event	Proteinuria or low eGFR		Proteinuria and low eGFR		
	Without proteinuria or low eGFR	Low eGFR alone	Proteinuria alone	Proteinuria following low eGFR	Low eGFR following proteinuria	Simultaneous proteinuria and low eGFR
Number	37276	3553	190	43	64	16
Women, %	33.4	25.5	16.3	11.6	12.5	6.3
Age, y	42 ± 9	44 ± 9	43 ± 8	46 ± 8 *	44 ± 8	44 ± 10
Height, cm	165.6 ± 5.8	166.4 ± 8.2	166.7 ± 7.4	167.8 ± 8.0	166.1 ± 6.6	166.2 ± 7.1
Weight, cm	62.3 ± 11.6	63.9 ± 10.6	68.1 ± 15.0***	67.0 ± 11.4*	68.7 ± 12.4***	73.8 ± 17.1
BMI, kg/m^2^	22.7 ± 3.4	23.0 ± 2.9	24.4 ± 4.5***	23.7 ± 2.9	24.8 ± 3.5***	26.5 ± 4.6*
FBS, mg/dL	98 ± 15	100 ± 13	110 ± 31***	106 ± 12***	111 ± 30**	127 ± 53
SBP, mmHg	120 ± 15	122 ± 16	129 ± 18***	133 ± 19***	131 ± 17***	135 ± 25
DBP, mmHg	72 ± 11	74 ± 10	77 ± 12***	79 ± 13**	79 ± 12***	81 ± 18
T-CHO, mg/dL	193 ± 33	194 ± 34	201 ± 34**	200 ± 37	201 ± 34	195 ± 27
TG, mg/dL	107 ± 75	119 ± 91	153 ± 106***	130 ± 65	149 ± 126*	165 ± 132
Follow-up Duration, y	9.5 ± 7.0	16.8 ± 7.3	15.5 ± 7.4***	19.7 ± 6.4***	19.5 ± 6.9***	15.9 ± 7.4*

Abbreviations: eGFR, estimated glomerular filtration rate; BMI, body mass index; FBS, fasting blood sugar; SBP, systolic blood pressure; DBP, diastolic blood pressure; T-CHO, total cholesterol; TG, triglyceride. The significance of differences in median values between no event and other subgroups was examined and is shown as ***P < 0.001, **P < 0.01, and *P < 0.05.

**Table 2.** Risk Factors for Renal Events with Proteinuria and Low eGFR.

Factor	Risk for renal outcomes, odds ratio (95% confidence interval)				
	Proteinuria or low eGFR		Proteinuria and low eGFR		
	Low eGFR alone (N = 3553)	Proteinuria alone (N = 190)	Proteinuria following low eGFR (N = 43)	Low eGFR following proteinuria (N = 64)	Simultaneous proteinuria and low eGFR (N = 16)
Sex, women	0.75 (0.69-0.81)***	0.54 (0.36-0.81)***	0.31 (0.12-0.88)*	0.41 (0.19-0.87)*	0.23 (0.03-1.75)
Age, +10 y	1.19 (1.14-1.23)***	1.00 (0.83-1.17)	1.47 (1.09-1.86)*	1.00 (0.72-1.30)	1.05 (0.50-1.63)
FBS, +10 mg/dL	1.00 (0.98-1.03)	1.14 (1.10-1.19)***	1.07 (0.93-1.21)	1.14 (1.07-1.22)***	1.23 (1.13-1.32)***
SBP, +10 mmHg	1.07 (1.03-1.11)**	1.31 (1.15-1.48)***	1.53 (1.22-1.85)**	1.42 (1.15-1.69)**	1.41 (0.89-1.96)
DBP, +10 mmHg	0.96 (0.90-1.02)	0.87 (0.63-1.12)	0.72 (0.26-1.19)	0.85 (0.45-1.26)	0.99 (0.21-1.84)
T-CHO, +10 mg/dL	0.99 (0.98-1.00)	1.01 (0.97-1.06)	1.02 (0.93-1.12)	1.02 (0.94-1.09)	0.93 (0.78-1.09)
TG, +10 mg/dL	1.01 (1.01-1.02)**	1.02 (1.01-1.03)***	1.00 (0.97-1.04)	1.01 (1.00-1.03)	1.02 (0.99-1.05)
Low BMI, <18.5 kg/m^2^	0.66 (0.55-0.79)***	1.10 (0.53-2.29)	0.61 (0.08-4.55)	0.48 (0.07-3.53)	nil
High BMI, >25.0 kg/m^2^	0.96 (0.88-1.05)	1.42 (1.02-1.96)*	0.85 (0.41-1.76)	1.99 (1.17-3.39)*	2.68 (0.93-7.75)

Risks for renal outcome were assessed by sex-, age- and other factor-adjusted logistic regression models. Reference range of BMI was 18.5-25.0 kg/m^2^. There were no subjects with low BMI with simultaneous proteinuria and low eGFR. The significance is shown as ***P < 0.001, **P < 0.01, and *P < 0.05. Abbreviations: eGFR, estimated glomerular filtration rate; BMI, body mass index; FBS, fasting blood sugar; SBP, systolic blood pressure; DBP, diastolic blood pressure; T-CHO, total cholesterol; TG, triglyceride.

Unfortunately, the detailed reasons for incident proteinuria, such as glomerulonephritis, nephrotic syndrome, and diabetic kidney disease, or the severity of proteinuria could not be investigated due to the study design. There was another limitation in that the health checkup cohort did not contain subjects with severe proteinuric CKD and end-stage renal disease because they were censored on follow-up and referred to a clinician. Therefore, hard outcomes such as renal death and cardiovascular death could not be assessed. Thus, whether obesity induces end-stage renal disease following persistent proteinuria based on these results remains unclear; investigations involving larger-sized cohorts with more consistent follow-up regardless of causes and severity of renal disease will be needed. A recent report from the Atherosclerosis Risk in Communities study with over 30 years of follow-up showed that obesity (hazard ratio per one-standard deviation greater BMI) is a significant risk factor for future decline in kidney function and development of end-stage renal disease, thus requiring renal replacement therapy in both black and white patients ^(6)^. Though they have successfully analyzed hard outcomes in a reliable cohort, the study completely lacks information regarding proteinuria, supposedly due to the health check system.

Different from other countries’ health check systems, the strength of Japan is that people of young age to elderly ones do an annual dipstick urine test to detect the early stage of chronic glomerulonephritis represented by immunoglobulin A nephropathy ^(7), (8)^. Consequently, this dipstick proteinuria may also be useful to detect cardiovascular disease risk group and, partially, diabetic kidney disease ^(9), (10)^. This present research may provide the impetus to use annual dipstick urine tests for health promotion to improve obesity in the Japanese general population. In this present study, we have also showed the different risks of aging and obesity for incident renal dysfunction and incident proteinuria, respectively, in a long-term follow-up of a cohort of health checkup visits over 30 years at maximum. The simple concept of “CKD” has become widespread and is useful for medical care by general practitioners, but it can mask the heterogeneity of CKD. Simultaneous confirmation of urinary protein and renal function might be a potential technique to identify obesity-related CKD.

## Article Information

### Conflicts of Interest

None

### Sources of Funding

This study was supported by the Japan Society for the Promotion of Science (JSPS) (grant no. #18KK0431 and #19K17729).

### Acknowledgement

The authors would like to thank Tomoko Takagi for contributing to data collection.

### Author Contributions

Conceptualization, Investigation, and Writing - Original Draft Preparation: Kei Nagai

Supervision and Writing - Review & Editing: Masami Nakajima, Shinichi Yoshii

### Approval by Institutional Review Board (IRB)

Ethics approval was obtained from the Institutional Review Board of Hitachi General Hospital (#2021-27).

### Informed Consent

Individual consent was not required for the analysis of this study, since it was conducted as a secondary use of data obtained for public health practice on disease prevention. Adhering to relevant guidelines and regulations afterward, participants were retrospectively given the opportunity to withdraw their data from analysis, and consent was considered to have been obtained if the participant did not decline.

### Data Availability

Data are available upon request to the corresponding author.
